# Electrochemical Immunoassay for Determination of Glycated Albumin using Nanozymes

**DOI:** 10.1038/s41598-020-66446-3

**Published:** 2020-06-11

**Authors:** Hyun Choi, Seong Eun Son, Won Hur, Van-Khue Tran, Han Been Lee, Yosep Park, Do Kyoung Han, Gi Hun Seong

**Affiliations:** 1 Department of Bionano Engineering, Hanyang University, Ansan, 426 – 791 South Korea; 2 Research Center for Materials Analysis, Korea Basic Science Institute, 169 – 148, Gwahak-ro, Yuseong-Gu, Daejeon, 34133 South Korea

**Keywords:** Biosensors, Nanoparticles

## Abstract

We developed a new nanozyme-based electrochemical immunoassay method for the monitoring of glycated albumin (GA) known to reflect short-term glycaemic levels. For this study, we synthesized urchin-like Pt nanozymes (uPtNZs) and applied them to colorimetric and electrochemical assays for sensitive determination of GA in total human serum albumin (tHSA) using 3,3′,5,5′-tetramethylbenzidine (TMB) and thionine as substrates, respectively. The uPtNZs showed peroxidase-mimic activity in the presence of hydrogen peroxide. Boronic acid (BA)-agarose bead was used to capture GA through specific cis-diol interactions. uPtNZs were modified with GA antibody (GA-Ab) to form sandwich complexes with GA/BA-agarose bead. The amount of Ab-uPtNZ/GA/BA-agarose bead complex increased with increasing percentage of GA in 50 mg/mL tHSA. The colorimetric assay exhibited linearity from 0.02 to 10% (10 µg/mL – 5 mg/mL) GA with an LOD of 0.02% (9.2 µg/mL). For electrochemical assay, GA was detected from 0.01 to 20% (5 µg/mL – 10 mg/mL) with an LOD of 0.008% (3.8 µg/mL). The recovery values of measured GA in human plasma samples were from 106 to 107%. These results indicate that electrochemical assay using uPtNZs is a promising method for determining GA.

## Introduction

Diabetes mellitus, which occurs worldwide, is a chronic metabolic disease with heterogenous etiologies resulting from insulin resistance and/or insulin deficiency^1^. It is characterized by an increased blood glucose concentration and causes a condition known as hyperglycemia^2^. Diabetes can lead to many complications such as nerve disease, kidney disease, peripheral arterial and cerebrovascular disease, and cardiovascular disease^3,4^. These diseases can be prevented by controlling and monitoring the level of glucose in the blood. There are several indicators of blood glucose concentration including 1,5-anhydro-D-glucitol (1,5-AG), glycated hemoglobin (HbA1c), and glycated albumin (GA). Since the supply, excretion, and metabolic rates of 1,5-AG are extremely low, 1,5-AG reflects blood glucose level from days to weeks ago. However, reduction in 1,5-AG level has been observed regardless of diabetes in patients with chronic renal failure or in pregnant women^5^. HbAlc is commonly used as the standard index of glycemic control and indicates plasma glucose level for the previous 2–3 months because its lifespan of is approximately 120 days^3^. However, due to the long lifespan of HbAlc, it is not appropriate for monitoring short-term blood glucose level fluctuations. In addition, HbAlc measurement is affected by diseases requiring hemodialysis or diseases in which erythrocyte lifespan is shortened^4^. Compared with other indicators, GA level sensitively reflects the average blood glucose level for the past 2–4 weeks and provides reliable readings of change in blood glucose level for patients with type 1 diabetes, chronic kidney disease, or those who are pregnant. The GA level is defined as the ratio of GA to total human serum albumin (tHSA; both glycated and non-glycated albumin) and is a good indicator of glycemia in diabetics who are self-monitoring^6^.

A number of methods can be employed to determine GA concentration, including colorimetric detection (e.g. thiobarbituric acid assay)^7,8^, enzymatic method^9^, affinity chromatography^10^, electrochemical analysis^11^, and high-performance liquid chromatography (HPLC)^12^. Among them, electrochemical analysis has many advantages for the clinical environment including fast data response, low cost, high sensitivity, and simplicity of operation^13–15^. By taking these advantages, enzymatic method-based electrochemical measurement of GA has been reported^11^. However, it still has the limitation for clinical applications because a natural enzyme is too unstable for reliable and accurate quantification of analyte.

The nanostructured artificial enzymes known as nanozymes are inspired by nature and aim to imitate the essential properties of natural enzymes using alternative materials^16^. Although natural enzymes have good catalytic activity and high substrate specificity, they are easily denatured and lose their activity under conditions such as certain ranges of pH and temperature. To deal with these problems, artificial enzymes have often been considered suitable alternatives to natural enzymes due to many advantages including ease of preparation, high stability, good cost-effectiveness, and excellent catalytic activity^17^. Based on these attractive and useful characteristics, nanozymes have been utilized in various applications such as biosensing, immunoassays, diagnostics, pollutant removal, and for clinical purposes^18,19^. Many efforts have been recently made to mimic the structures and functions of natural enzymes through diverse approaches with various structures and different compositions using carbon materials, noble metals, polymers, and metal oxides^20–22^. Among them, platinum-based nanoparticles (PtNPs) have been extensively utilized in various shapes or as composites with other metals because PtNPs exhibit impressive peroxidase-mimicking activity with high stability and sensitivity^23–27^. Previous reports have shown that PtNPs used as peroxidase mimics oxidized various substrates such as 3,3′,5,5′-tetramethylbenzidine (TMB), 3-amino-9-ethyl carbazole (AEC), and luminol in the presence of hydrogen peroxide (H_2_O_2_)^28–30^.

**Figure 1:**
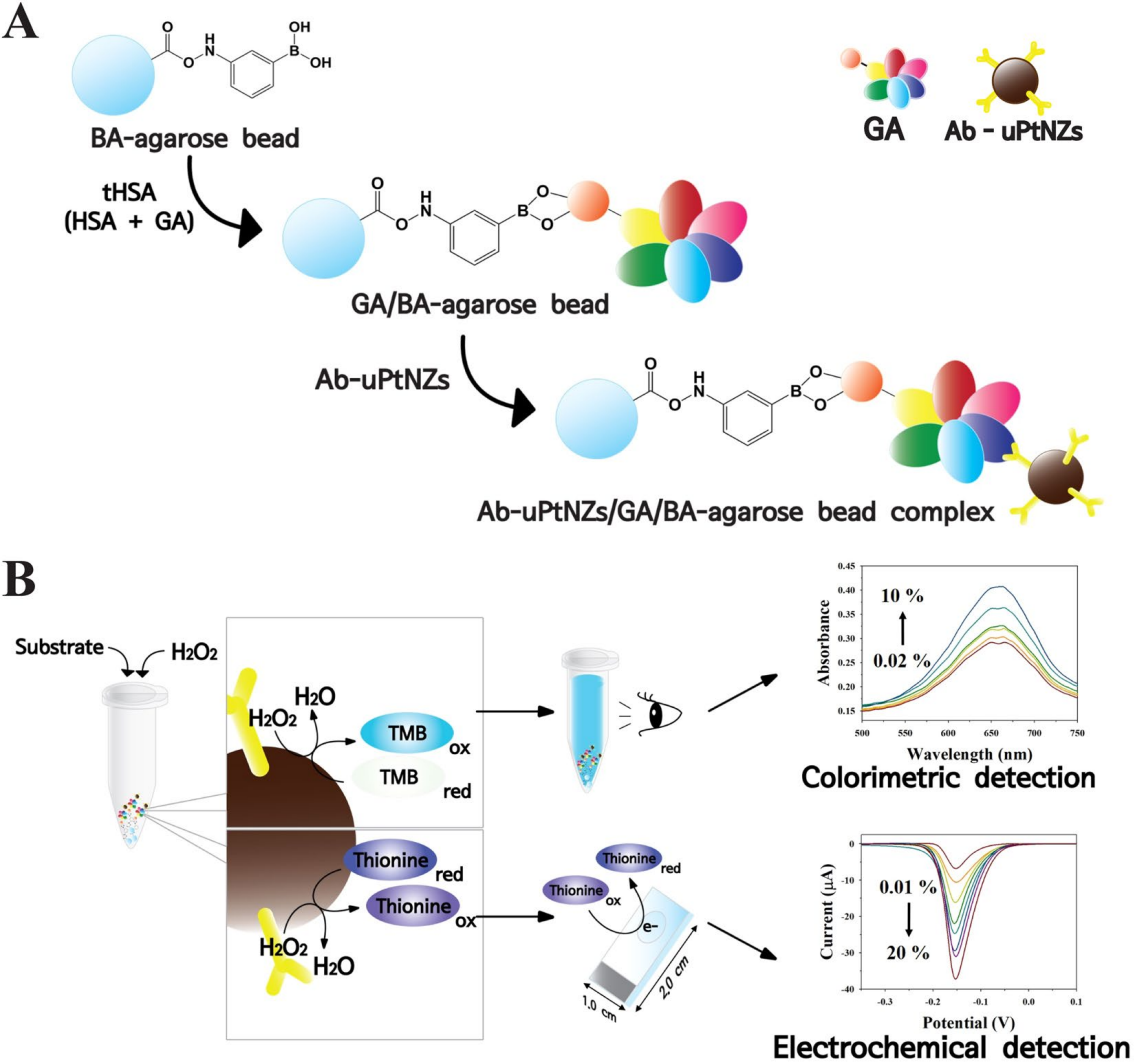
Experimental diagram of (**A**) preparation of Ab-uPtNZ/GA/BA-agarose bead complex and (**B**) colorimetric and electrochemical detection of GA using uPtNZs. Ab-uPtNZ/GA/BA-agarose bead complexes were formed through the boronic acid-cis-diol interaction and the sandwich immunoreaction. The prepared complexes were incubated with TMB chromogenic substrate and thionine electrochemical mediator, and then resulting colored and electrochemical products were analyzed by colorimetric and electrochemical detection, respectively.

In this study, we developed an electrochemical immunoassay for sensitive detection of GA using uPtNZs. Due to the high surface ratio of uPtNZs, they exhibited highly efficient peroxidase-like activity toward substrates in the presence of H_2_O_2_. The assay was performed with BA-agarose bead as GA capture probes. These well-coordinated agarose beads allowed selective detection of GA through boronic acid-cis-diol interactions of the sugars. In GA detection, sandwiched complexes were formed between the BA-agarose bead and anti-human serum glycated albumin antibody labeled uPtNZ (Ab-uPtNZ) (Fig. 1A). uPtNZs then catalyzed substrates in the presence of H_2_O_2_. TMB and thionine were used as substrates in colorimetric and electrochemical methods, respectively (Fig. 1B). Both methods showed specific peaks in absorbance spectroscopy and differential pulse voltammetry (DPV), and these results were used to quantify the concentration of GA. Moreover, the electrochemical immunoassay was evaluated by determining GA in human plasma samples using spike recovery and was compared with results from a standard ELISA method.

## Results and Discussion

**Figure 2:**
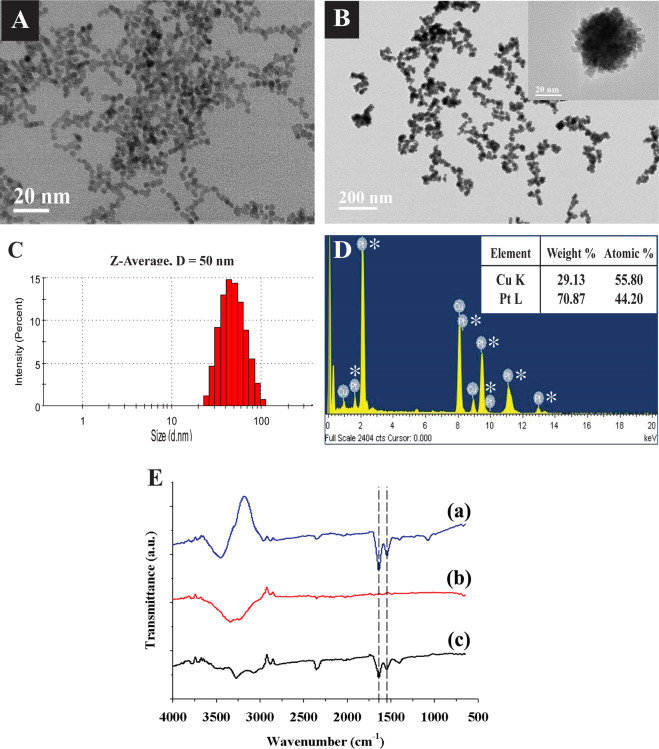
(**A**) TEM images of Pt seeds suspensions and (**B**) uPtNZs suspensions. (**C**) DLS size distribution and (**D**) EDX spectra of uPtNZs (*represent Pt peaks). (**E**) FT-IR spectra of (a) GA-Ab, (b) bare uPtNZs, and (c) Ab-uPtNZs.

The characteristics of uPtNZs and Ab-uPtNZs are shown in Fig. 2. Figure 2A shows well synthesized Pt seeds with a diameter of ~5 nm. uPtNZs exhibited fairly uniform dispersion with a mean diameter of ~40 nm in TEM images (Fig. 2B). However, DLS indicated that the mean size of uPtNZs was 50 nm (Fig. 2C). While TEM measures particle core size, DLS measures particle hydrodynamic diameter (core size + diffuse layer)^31^. Thus, the size measured by DLS is generally larger than that measured by TEM. The concentration of uPtNZs was measured by ICP-AES to be 0.316 ± 0.036 mg/mL at 306 nm. uPtNZs were also investigated by energy-dispersive X-ray (EDX) spectroscopy, and a Pt peak (44.20 atomic %) was obtained (Fig. 2D), along with a peak of Cu due to the background grid. These results proved successful synthesis of uPtNZs.

uPtNZs were modified with GA-Ab for GA binding. The FT-IR spectra of uPtNZs before and after conjugation with antibodies were investigated. As is well known, proteins have strong IR absorption peaks around 1650 cm^−1^ and 1550 cm^−1^ corresponding to the peptide group^32,33^. As shown in Fig. 2E, two obvious peaks at ~1640 cm^−1^ and ~1560 cm^−1^ were exhibited for antibodies (curve a), while no specific peak was observed from 2800 cm^−1^ to 650 cm^−1^ for uPtNZs (curve b). After antibodies were conjugated on the surface of uPtNZs, the characteristic peaks for the peptide group were recorded (curve c). These results indicate that Ab-uPtNZs were successfully formed with physical adsorption by hydrophobic interaction and electrostatic attraction.

**Figure 3:**
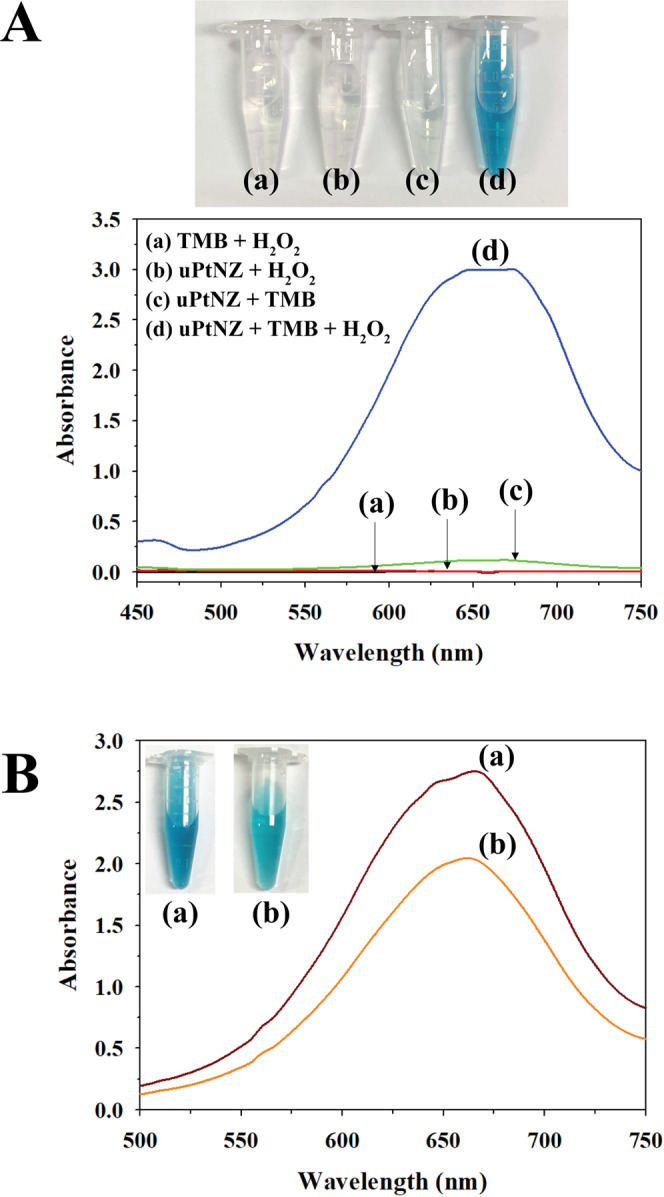
(**A**) Photographs of color change and UV-vis spectra of (a) TMB + H_2_O_2_ (black), (b) uPtNZs + H_2_O_2_ (red), (c) uPtNZs + TMB (green), and (d) uPtNZs + TMB + H_2_O_2_ (blue). (**B**) Differences in peroxidase activity between (a) bare uPtNZs and (b) Ab-uPtNZs with 0.5 mM TMB and 80 mM H_2_O_2_.

We investigate peroxidase-like activity of uPtNZs. Upon the addition of uPtNZs to the solution containing TMB and H_2_O_2,_ the TMB color change was clearly observed within 1 min, and the UV-vis spectra of blue TMB_ox_ showed strong absorption at 652 nm (Fig. 3A). These results indicate that TMB was oxidized successfully by the oxidase-mimicking activity of uPtNZs. The nanozymes also showed slight color change without H_2_O_2_, but this oxidase-mimic activity was weak enough to ignore. When antibodies were conjugated on uPtNZs, the peroxidase-like activity decrease slightly but remained adequate for use as nanozymes (Fig. 3B).

We further investigated the catalytic activity of uPtNZs using enzyme kinetic analysis with H_2_O_2_ and TMB. This analysis was carried out using time-dependent absorbance at 652 nm to measure the rate of TMB_ox_ production catalyzed by uPtNZs (Supplementary Fig. S1A and S1B). Michaelis-Menten curves were obtained within the appropriate ranges of H_2_O_2_ and TMB (Supplementary Fig. S1C,D). The curves were plotted using initial velocity (*V*_0_) of TMB_ox_ formation dependent on substrate concentration ([*S*]). The curves were saturated at 100 mM H_2_O_2_ and 0.5 mM TMB, and these concentrations were used for further experiments. Then, the data were fitted to the Lineweaver-Burk reciprocal plot to obtain the Michaelis-Menten constant (*K*_m_) and maximum velocity (*V*_max_), which are important enzyme kinetic parameters (Supplementary Fig. S1E,F). Table 1 shows the kinetic parameters of uPtNZs and various peroxidase-mimicking enzymes. The *K*_m_ value toward TMB of the uPtNZs is 0.174 mM, while the *K*_m_ toward H_2_O_2_ is 82.7 mM. The lower is the *K*_m_ value, the higher is the affinity with a substrate^34^. Therefore, uPtNZs have a higher affinity toward TMB and H_2_O_2_ substrate compared to most nanozymes. Furthermore, uPtNZs can react faster than horseradish peroxidase (HRP) leading to a higher catalytic constant (*k*_*cat*_), which means that they have higher catalytic activity than HRP and can be used as an alternative to HRP.

**Table 1:** Comparison of uPtNZs with other peroxidase-mimicking nanoparticles.

Sample	[E] (M)	Substrate	Km (mM)	V_max_ (µM s^−1^)	kcat (s^−1^)	Ref.
HRP	1.0 × 10^−9^	TMB	0.147	1.00 × 10^−1^	7.90 × 10^2^	^43^
		H_2_O_2_	0.146	8.70 × 10^−2^	7.82 × 10^2^	
Fe_3_O_4_	7.9 × 10^−9^	TMB	0.233	1.76 × 10^−1^	2.22 × 10^1^	^44^
		H_2_O_2_	479.91	2.75 × 10^−1^	3.47 × 10^1^	
Co_3_O_4_	2.5 × 10^−9^	TMB	0.103	2.56 × 10^−1^	1.01 × 10^2^	^44^
		H_2_O_2_	173.51	1.89 × 10^−1^	7.47 × 10^1^	
MnO_2_	3.0 × 10^−8^	TMB	0.04	5.78	1.92 × 10^2^	^45^
		H_2_O_2_	0.12	5.71× 10^−2^	1.90 × 10^1^	
RuO_2_	3.5 × 10^−11^	TMB	0.236	1.90 × 10^−1^	5.43 × 10^3^	^21^
		H_2_O_2_	212	2.05× 10^−1^	5.86 × 10^3^	
PBNP^a^	6.7 × 10^−12^	TMB	0.76	1.74 × 10^−1^	2.60 × 10^4^	^46^
		H_2_O_2_	840	1.27 × 10^−1^	1.90 × 10^4^	
uPtNZs	3.7 × 10^−11^	TMB	0.174	1.01	2.69 × 10^4^	This study
		H_2_O_2_	82.7	1.77	4.75 × 10^4^	

^a^PBNP: Prussian blue nanoparticle.

**Figure 4:**
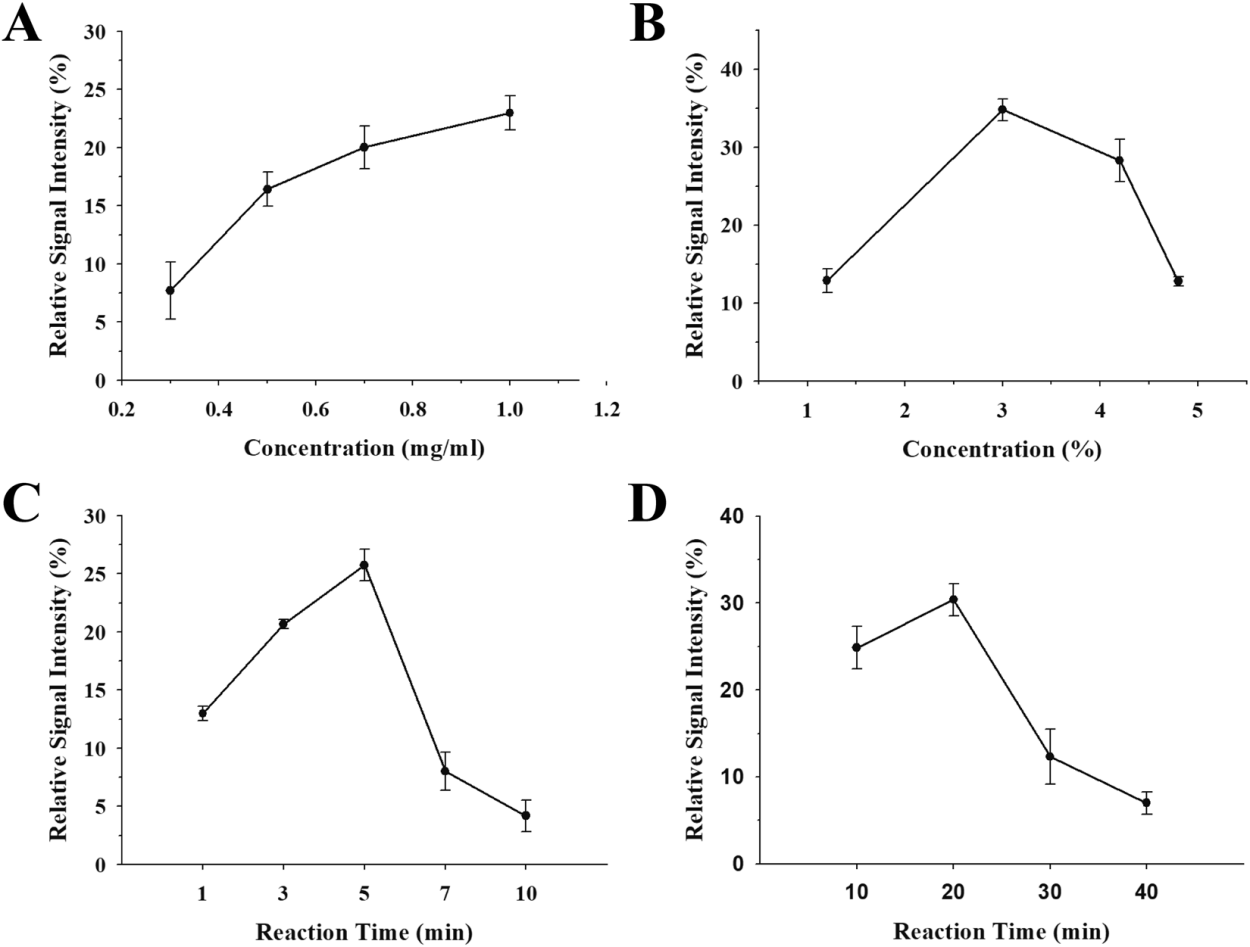
The impact of (**A**) antibody concentration, (**B**) BA-agarose bead concentration, and (**C**) reaction time between BA-agarose bead and GA in tHSA and (**D**) between GA/BA-agarose bead and Ab-uPtNZs on the efficiency of immunoassay with 10% GA in 50 mg/mL tHSA, 0.5 mM TMB, and 100 mM H_2_O_2_. In each case, other variables were controlled to a fixed condition.

To obtain high efficiency in the immunoassay, the effects of antibody concentration, BA-agarose bead amount, and reaction time for formation of Ab-uPtNZ/GA/BA-agarose bead complex was investigated by measuring the change in absorption intensity of TMB_ox_. The intensity of TMB_ox_ increased with increasing antibody concentration up to 1.0 mg/mL (Fig. 4A). At over 1.0 mg/mL of the antibody, Ab-uPtNZs were observed to aggregate after centrifugation. When the percentage of agarose bead exceeded 3%, the blank intensity increased because of an increase in non-specific entrapment of Ab-uPtNZs in agarose beads, resulting in decrease of the relative intensity of TMB_ox_ to the blank (Fig. 4B). Thus, we chose 1.0 mg/mL of the antibody and 3% agarose bead solution as optimized conditions. The process of Ab-uPtNZ/GA/BA-agarose bead complex formation has two steps (Fig. 1A); the first step is the reaction between BA-agarose beads and GA, in which boronic acid (BA) interact with the sugar groups in GA through a cis-diol binding reaction known as boronate affinity^35,36^. The second step is immunoreaction between GA/BA-agarose bead and Ab-uPtNZs. The highest intensity was obtained when reaction times for the first and second steps were set to 5 and 20 min, respectively (Fig. 4C,D). Intensity increased as reaction time increased and then decreased as the time became too long. Too long of a reaction time offered opportunity for non-specific entrapment and resulted in a higher blank signal. Therefore, the optimum conditions of 1.0 mg/mL antibody, 3% BA-agarose beads, and respective 5 and 20 min of reaction time for the first and second steps were chosen for further experiments.

**Figure 5:**
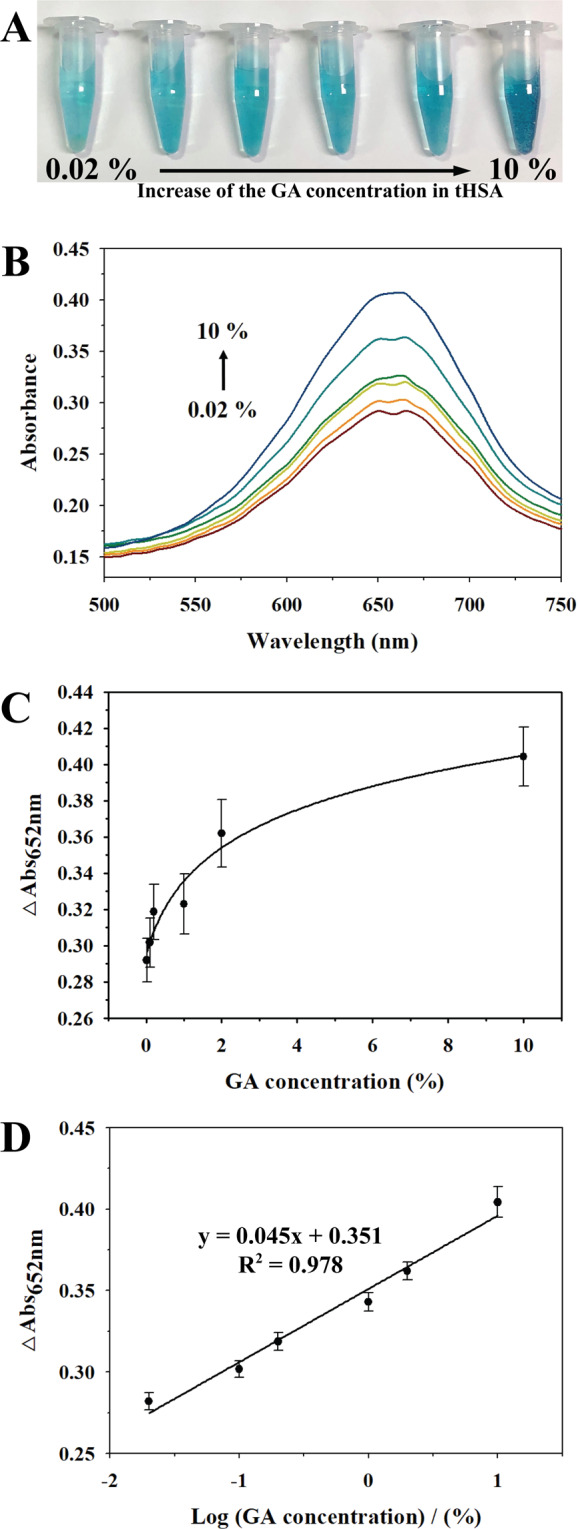
(**A**) Photographs and (**B**) UV-vis absorbance spectra of 0.5 mM TMB with uPtNZs and 100 mM H_2_O_2_ at different concentrations of GA (0.02, 0.1, 0.2, 1, 2, 10%) in 50 mg/mL tHSA. (**C**) Colorimetric calibration curve for GA detection. (**D**) Linear relationship between absorbance of TMB_ox_ and GA logarithmic concentration.

To evaluate analytical performances using uPtNZs for the detection of GA, the colorimetric assay was carried out by varying the concentration of GA under optimized conditions. The blue color of TMB_ox_ became deeper as GA concentration increased (Fig. 5A). The absorbance intensities of TMB_ox_ at 652 nm gradually increased with increasing concentration of GA (Fig. 5B). Figure 5C,D show the colorimetric calibration curves for different GA % in tHSA, and a good linear relationship between absorbance of TMB_ox_ and GA logarithm concentration was obtained in the detection range of 0.02–10% (10 µg/mL – 5 mg/mL) GA with a correlation coefficient (R^2^) of 0.978 (*n* = 3). A limit of detection (LOD) of 0.02% (9.2 µg/mL) was calculated by three times the standard deviation of blanks.

**Figure 6:**
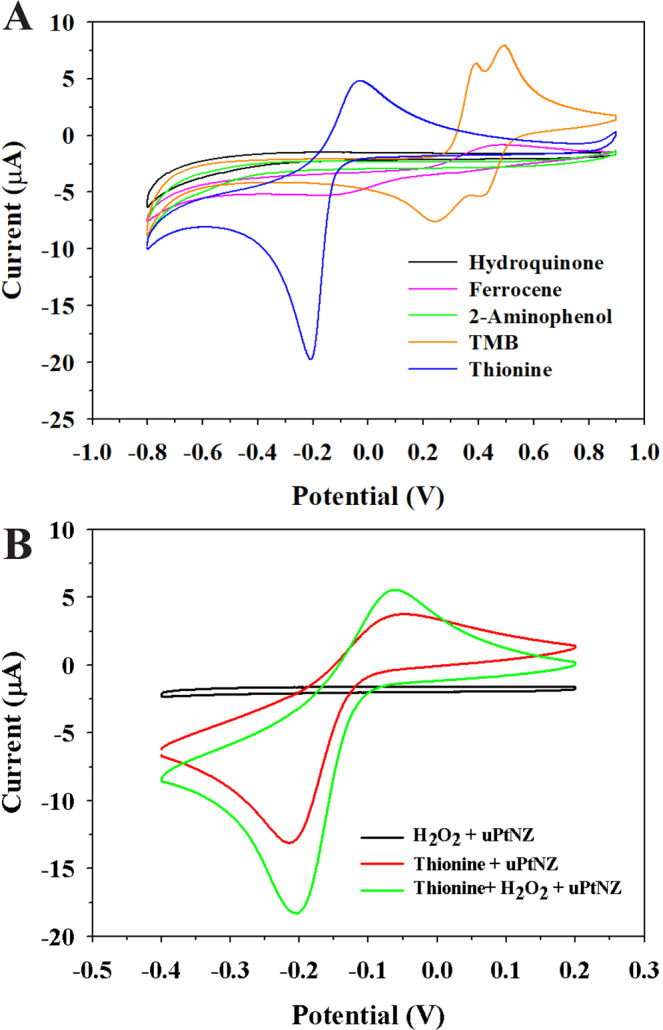
(**A**) CV spectra of 0.5 mM of various substrates (hydroquinone, ferrocene, *o*-aminophenol, TMB, thionine) in the presence of uPtNZs and 100 mM H_2_O_2_ at a scan rate of 100 mV/s. (**B**) CVs of the peroxidase activity of uPtNZs with 0.5 mM thionine and 100 mM H_2_O_2_.

We applied uPtNZs to electrochemical assay under the same optimized condition as that of the colorimetric assay. To achieve a high-sensitivity electrochemical assay, several substrates known as electrochemical mediators of the HRP were compared. In the cyclic voltammograms (CVs), the reduction peak of thionine showed the highest signal compared to other substrates in PBS (pH 7.4) (blue curve in Fig. 6A). The oxidation/reduction potential of thionine was less positive than that of others, which is more favorable for avoiding the potential interference from interfering competitive species. Under the same condition, thionine has more aromatic ring structure than other substrates, so it has good electrical conductivity due to the large number of delocalized electrons^37^. In addition, thionine has a sulfur atom which is less electronegative than oxygen or nitrogen, and sulfur π-electrons are more delocalized. Moreover, thionine has the high activity at pH 7.4, which is useful to apply electrochemical assay using thionine to real samples^38,39^. In the CVs of Fig. 6B, an obvious increase of the reduction peak of thionine was observed when uPtNZs and H_2_O_2_ were present. This result indicated that uPtNZs have good peroxidase-mimic activity for thionine.

**Figure 7:**
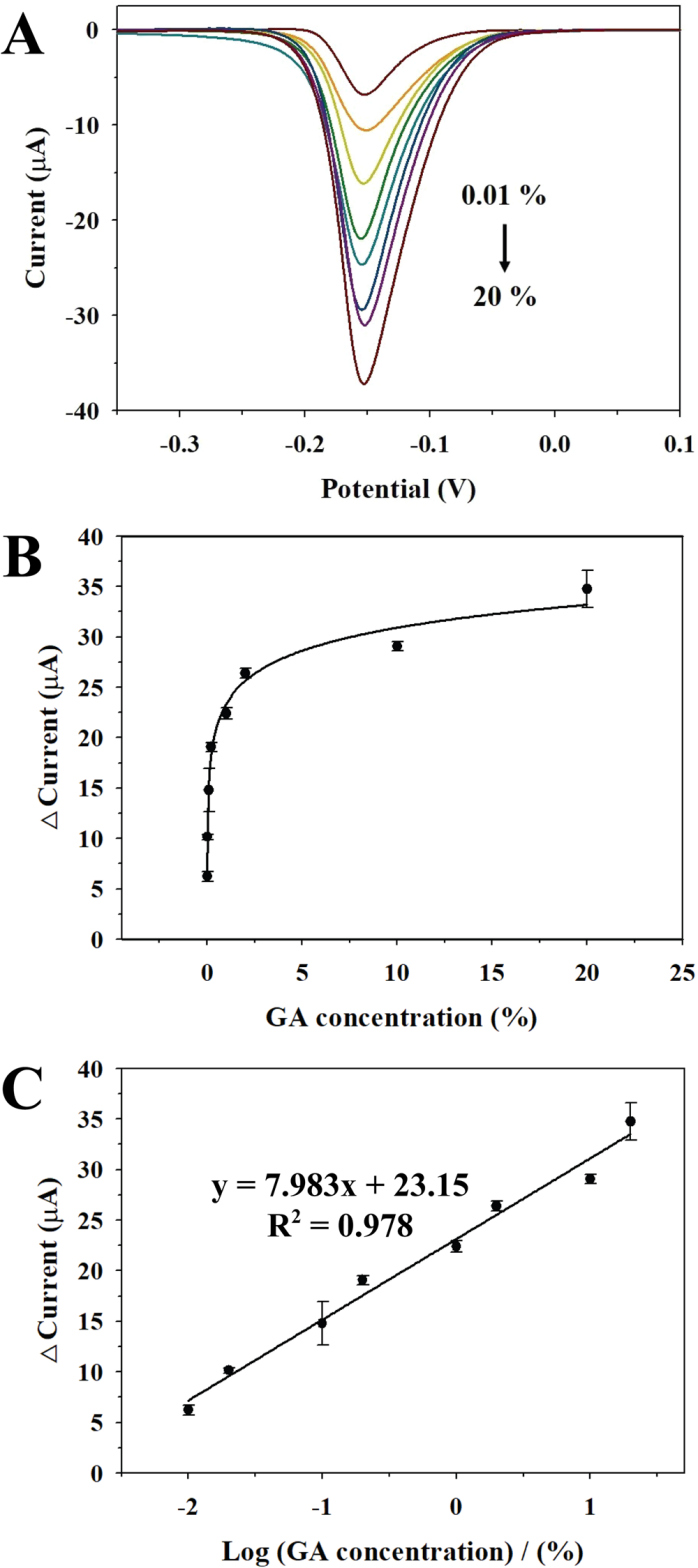
(**A**) DPV spectra of 0.5 mM thionine with uPtNZs and 100 mM H_2_O_2_ at different concentrations of GA (0.01, 0.02, 0.1, 0.2, 1, 2, 10, 20%) in 50 mg/mL tHSA. (**B**) Electrochemical calibration curve for GA detection. (**C**) Linear relationship between reduction current of thionine_ox_ and GA logarithmic concentration.

Thus, electrochemical determination of different concentrations of GA in 50 mg/mL tHSA was carried out using DPV (amplitude 50 mV; pulse width 0.05 s; pulse period 0.5 s) in the presence of 0.5 mM thionine and 100 mM H_2_O_2_. The parameters of DPV were optimized as shown in Supplementary Fig. S2. To compare with colorimetric assay, the concentration of thionine fixed to 0.5 mM same as TMB. The current peaks increased with increasing GA concentration at −150 mV (Fig. 7A). Figure 7B shows the electrochemical calibration curve between reduction current of thionine_ox_ and concentration of GA. The GA concentration was converted to a logarithmic scale to obtain a linear relationship with current difference (Fig. 7C). A linear relationship was observed in the range of 0.01–20% (5 µg/mL – 10 mg/mL) GA with a correlation coefficient (R^2^) of 0.984 (*n* = 3). The GA was detected as low as 0.008% (3.8 µg/mL), which was lower than the LOD of the colorimetric assay. These results show that the electrochemical assay for GA determination with uPtNZs has a broader detection range, a lower LOD, and higher sensitivity compared to colorimetric assay.

The quantification of tHSA is an indispensable step for GA assay because GA level is determined by getting the proportion of GA to tHSA. The reaction between albumin and BCG dye was used to measure the concentration of tHSA. For this, the concentration of BCG was optimized first. It was found that the 1.0 mM BCG solution was most appropriate because the relative intensity of the blue color of BCG-albumin complexes to the green color of excess BCG was saturated (Supplementary Fig. S3A). The color of the solution changed from green to blue with increasing concentration of tHSA. The calibration curve between the relative signal intensity of blue color to green color and tHSA concentration was obtained (Supplementary Fig. S3B) and showed a good linear relationship in the detection range of 0.5–50 mg/mL (R^2^ = 0.981, *n* = 3), with an LOD of 71.8 µg/mL. With a combination of both tHSA and GA detection methods, the determination of GA level can be achieved by calculating the percentage of GA relative to tHSA.

Accuracy of the electrochemical immunoassay was evaluated by determining GA in human plasma samples using spike recovery and was compared with the values found by the standard ELISA method. Different amounts of GA were added to human plasma and analyzed by our electrochemical immunoassay and a commercial ELISA kit (detection range: 1.30–83.2 µg/mL). As indicated in Table 2, the recovery values of the electrochemical method ranged from 106% to 107%, very similar to those found by the ELISA kit, which laid within the acceptable recovery range of 80–110% based on FDA guidelines for the validation of chemical methods^40^. The relative percentage difference (RPD) was calculated to confirm the accuracy of our assay for GA measurement and was less than 1%. Moreover, analytical performance of our device is comparable to or better than those from other methods described in literatures (Supplementary Table S1). These results demonstrate the reliability and feasibility of our method for GA analysis without significant interference in real sample matrix.

**Table 2:** Determination of GA in human plasma sample with a commercial ELISA kit and electrochemical assay.

Added (µg/mL)	ELISA measurement		Electrochemical assay		RPD (%)
	Found	Recovery (%)	Found	Recovery (%)	
10	10.35	103.5	10.74	107.4	0.92
50	51.58	103.16	52.98	105.96	0.67

## Conclusion

The electrochemical/colorimetric immunoassay for GA determination was developed using uPtNZs. Kinetic studies of uPtNZs showed excellent peroxidase-like activity compared with other nanozymes, which then was used to detect GA using colorimetric and electrochemical measurements. For the detection of GA, uPtNZs were modified with GA-Ab. Nanozyme complexes were formed between BA-agarose beads, GA, and Ab-uPtNZs at different concentrations of GA. Then, nanozyme complexes strongly oxidized TMB or thionine with H_2_O_2_ within one minute. These results were analyzed by UV-vis spectroscopy and DPV, respectively. The colorimetric method showed linearity over the range of 0.02–10% (10 µg/mL – 5 mg/mL) GA with an LOD of 0.02% (9.2 µg/mL). The electrochemical method presented a linear relationship in the range of 0.01–20% (5 µg/mL – 10 mg/mL) GA with an LOD of 0.008% (3.8 µg/mL), which cover the range of GA concentration in healthy plasma (0.21–1.65 mg/mL)^35^. The results demonstrate that the electrochemical immunoassay was more sensitive for detection of GA compared to the colorimetric method. For the determination of GA level in tHSA, colorimetric tHSA assay was performed in combination with electrochemical GA assay. BCG-albumin colored complexes were used for the detection of tHSA. There was a linear correlation between color intensity of complexes and tHSA over the range of 0.5–50 mg/mL and as low as 71.8 µg/mL. In addition, the accuracy of the electrochemical immunoassay was evaluated using a recovery test and compared to that of the ELISA kit. The recovery values ranged from 106% to 107% and were very similar to ELISA results. Based on these results, we expect that the electrochemical immunoassay for GA using uPtNZs can be a useful tool for facilitating self-monitoring of diabetes and a number of immunoassays applications.

## Materials and Methods

### Materials

Chloroplatinic acid hydrate (H_2_PtCl_6_), sodium hydroxide (NaOH), sodium citrate, sodium borohydride (NaBH_4_), L-ascorbic acid (AA), 30 K polyvinylpyrrolidone (PVP), sodium azide (NaN_3_), sucrose, sodium chloride (NaCl), TMB, thionine acetate salt, H_2_O_2_, HSA, GA, human plasma, BA-agarose bead, indium tin oxide (ITO) film, bromocresol green (BCG), and succinic acid were purchased from Sigma-Aldrich (St. Louis, MO, USA). The GA antibody (mouse anti-HSA, glycated monoclonal antibody) were obtained from MyBioSource (San Diego, CA, USA). The GA ELISA kit was supplied from LSBio (Seattle, WA, USA). The phthalate buffer (pH 4.0) and phosphate-buffered saline (PBS, pH 7.4) were provided by Samchun (Pyeongtaek, South Korea) and Biosesang, Inc. (Sung Nam, South Korea), respectively. Polymeric film was purchased from Two-Hand Co., Ltd (Anseong, South Korea). All other reagents were of analytical grade, and aqueous solutions were prepared with deionized (DI) water.

### Synthesis of uPtNZs

For synthesis of uPtNZs, Pt seeds were prepared^31,32^. For this, 2.16 mL of 5 mM H_2_PtCl_6_ was added to 27.84 mL of DI water in an 80 °C water bath. Then, 150 µL of 0.02 M NaOH was added to produce monodisperse, small PtNPs. After 1 min, 660 µL of 1% sodium citrate solution was added dropwise. After 30 s, 330 µL of 1% sodium citrate and 0.08% NaBH_4_ were added quickly to the mixture under constant stirring. After 10 min, the solution was left to cool to room temperature. The product was purified two times by centrifugation with DI water at 15,000 rpm. Finally, to prepare uPtNZs, 1 mL Pt seeds suspension was diluted in 29 mL DI water, and 45 µL of 0.4 M H_2_PtCl_6_ was added under constant stirring. Then, 500 µL of 1% sodium citrate and 1.25% AA solution were added dropwise to the mixture. The temperature was slowly raised from 60 °C to 80 °C, and the solution was incubated in an 80 °C water bath for 30 min. The resultant solution was left to cool to room temperature and then purified by centrifugation with DI water. The prepared uPtNZs were resuspended in DI water and stored at 4 °C before use.

### Preparation of Ab-uPtNZs solution

To prepare Ab-uPtNZs^33^, 10 µL of 1 mg/mL GA-Ab was added to uPtNZs solution (0.15 mg/mL), and then 10 µL of 0.1 M NaOH was added. After incubation for 5 h at 4 °C, 50 µL of 2% PVP in PBS was added to the solution to block non-specific adsorption on the Pt surface. After incubation for 5 h at 4 °C, the mixture was purified by centrifugation with DI water at 13,500 rpm. The supernatant was discarded, and Ab-uPtNZs was resuspended in PBS buffer (pH 7.4) containing 0.2% PVP, 0.02% NaN_3_, and 1% sucrose and stored at 4 °C until further use.

### Kinetic assay of uPtNZs

The time-dependent kinetic assay was performed to detect the peroxidase-like activity of uPtNZs^41^. The concentrations of H_2_O_2_ and TMB were fixed at 100 mM and 0.5 mM, respectively, in phthalate buffer (pH 4). Then, 30 µL of uPtNZs (0.019 mg/mL) was added to the solution (970 µL) to initiate the oxidation reaction. Initial velocity (*V*_0_) was determined according to Eq. (a,b):

a $$C=\frac{{A}_{652{\rm{nm}}}}{\varepsilon L}$$

b $${V}_{0}=\frac{\Delta C}{\Delta t}$$

where *C* represents the concentration of TMB_ox_, *ɛ* is the extinction coefficient of TMB_ox_ (3.9 × 10^4^ M^−1^cm^−1^), and *L* is the optical path length of the cuvette (1 cm).

The enzymatic parameters of nanozymes were calculated using the Michaelis-Menten equation (Eq. (c)) and the Lineweaver-Burk plot method (Eq. (d)):

c $${V}_{0}=\frac{{V}_{\max }[S]}{{K}_{{\rm{m}}}+[S]}$$

d $$\frac{1}{{V}_{0}}=\frac{{K}_{{\rm{m}}}}{{V}_{\max }[S]}+\frac{1}{{V}_{{\max }}}$$

where *V*_max_ is the maximum velocity, [S] is the substrate concentration, and *K*_m_ is the Michaelis-Menten constant.

### Preparation of electrochemical device

ITO-coated film (10 × 20 mm) with 140 μm thickness was prepared as a working electrode strip. The reaction window with diameter of 6 mm was designed using AutoCAD and the adhesive plastic film (160 μm thickness) was cut according to the window design with a laser cutter (Micro Laser Machine C40, Coryart). The cut adhesive film was placed on the ITO film to make blocking layer and specific detection area. Then, the device was laminated to ensure the complete adhesion and durable stability. The total cost for the fabrication of device is estimated at 5 cents, thereby allowing the cost-effective assay for single-use test. The prepared devices can be stored at room temperature, where they are stable for two months.

### Colorimetric and electrochemical assays of GA

For colorimetric and electrochemical quantification of GA, Ab-uPtNZ/GA/BA-agarose bead complex was formed (Fig. 1A). For this, 40 µL of BA-agarose beads and 10 µL of various percentages of GA (in 50 mg/mL of tHSA) in PBS buffer (pH 7.4) containing 0.9% NaCl and 0.05% NaN_3_ were incubated at room temperature. After incubation, the GA/BA-agarose beads (~50 µm) were washed by three cycles of centrifugation at 6,000 rpm for 10 s in PBS solution. Then, 10 µL of Ab-uPtNZs conjugates (~40 nm) were added to the GA/BA-agarose bead. After incubation at room temperature, excess conjugates were removed with centrifugation at 6,000 rpm for 5 s. For this, the optimized conditions of the reaction among BA-agarose bead, GA, and Ab-uPtNZs were investigated under different GA-Ab concentrations, BA-agarose bead amounts, and reaction time. Then, colorimetric and electrochemical assays were carried out with these Ab-uPtNZ/GA/BA-agarose bead complexes (Fig. 1B).

The colorimetric assay was performed first, for which 0.5 mM TMB and 100 mM H_2_O_2_ in phthalate buffer (pH 4) were added to the complexes. After incubation for 1 min at room temperature, the absorbance of TMB_ox_ was monitored using UV-vis spectroscopy. For electrochemical assay, 0.5 mM thionine and 100 mM H_2_O_2_ in PBS (pH 7.4) were added to the Ab-uPtNZ/GA/BA-agarose bead complex. After incubation for 1 min at room temperature, DPV of the reduction of thionine_ox_ were performed using an electrochemical analyzer (CH Instruments Inc., CHI660E). In a three-electrode setup for electrochemical assay, an ITO film electrode was used as the working electrode (WE), and Ag/AgCl (saturated in 3 M NaCl) and Pt wire were used as the reference electrode (RE) and the counter electrode (CE), respectively. To produce a specific detection area on the ITO electrode, a transparent adhesive polymeric film with a 3.0 mm diameter hole was attached.

### Quantification of tHSA with BCG

The well-known albumin detection method in which albumin-BCG complexes form at pH 4.2 instigates a color change from green to blue because of the specific interaction between albumin and BCG^42^. For this assay, the complexes were generated by adding 20 µL of different concentrations of tHSA (4% GA) to 60 µL of 1 mM BCG in a 0.2 M succinic acid buffer (pH 4.2). After 1 min, the solution was adjusted to 100 µL final volume, and the absorbance of the BCG at 625 nm was monitored using a Synergy Mx multimode microplate reader from Biotek (Winooski, VT, USA).

## Supplementary information

Supplementary information.
