# Supplementary material for: Electrochemical Immunoassay for Determination of Glycated Albumin using Nanozymes
Source: Sci Rep. 2020 Jun 11;10:9513. doi: 10.1038/s41598-020-66446-3 (PMC7289889; doi:10.1038/s41598-020-66446-3)
Supplement: Supplementary file 1 — Supplementary information. [file 41598_2020_66446_MOESM1_ESM.pdf]

Electrochemical Immunoassay for Determination of Glycated Albumin using Nanozymes

Hyun Choi[a], Seong Eun Son[a], Won Hur[a], Van-Khue Tran[a], Hanbeen Lee[a],

Yosep Park[a], Do Kyoung Han[b,]*, and Gi Hun Seong[a,]*

[a]Department of Bionano Engineering, Hanyang University, Ansan 426–791, South Korea

[b]Research Center for Materials Analysis, Korea Basic Science Institute, 169–148, Gwahak-ro, Yuseong-Gu, Daejeon, 34133, South Korea

*Corresponding author:

Gi Hun Seong: ghseong@hanyang.ac.kr

Do Kyoung Han: knhan3725@kbsi.re.kr

Supplementary Information

Supplementary Method

Characterization of uPtNZs and Ab-uPtNZs

The concentration of uPtNZs stock solution was determined using inductively coupled plasma atomic emission spectroscopy (ICP-AES, SPECTRO ARCOS, Germany). The size distribution of uPtNZs was confirmed using dynamic light scattering (DLS, Malvern Nano ZS90, UK). The morphologies of Pt seeds and uPtNZs were characterized by transmission electron microscopy (TEM, JEOL JEM-2010, Japan). Ab-uPtNZs were demonstrated by Fourier-transform infrared (FT-IR) spectroscopy on a Nicolet iS50 FTIR spectrometer (Thermo Fisher Scientific, USA). Ultraviolet-visible (UV-vis) absorption spectra were produced with an OPTIZEN 2120UV spectrometer (Mecasys Co. Ltd., South Korea).

Supplementary Figures

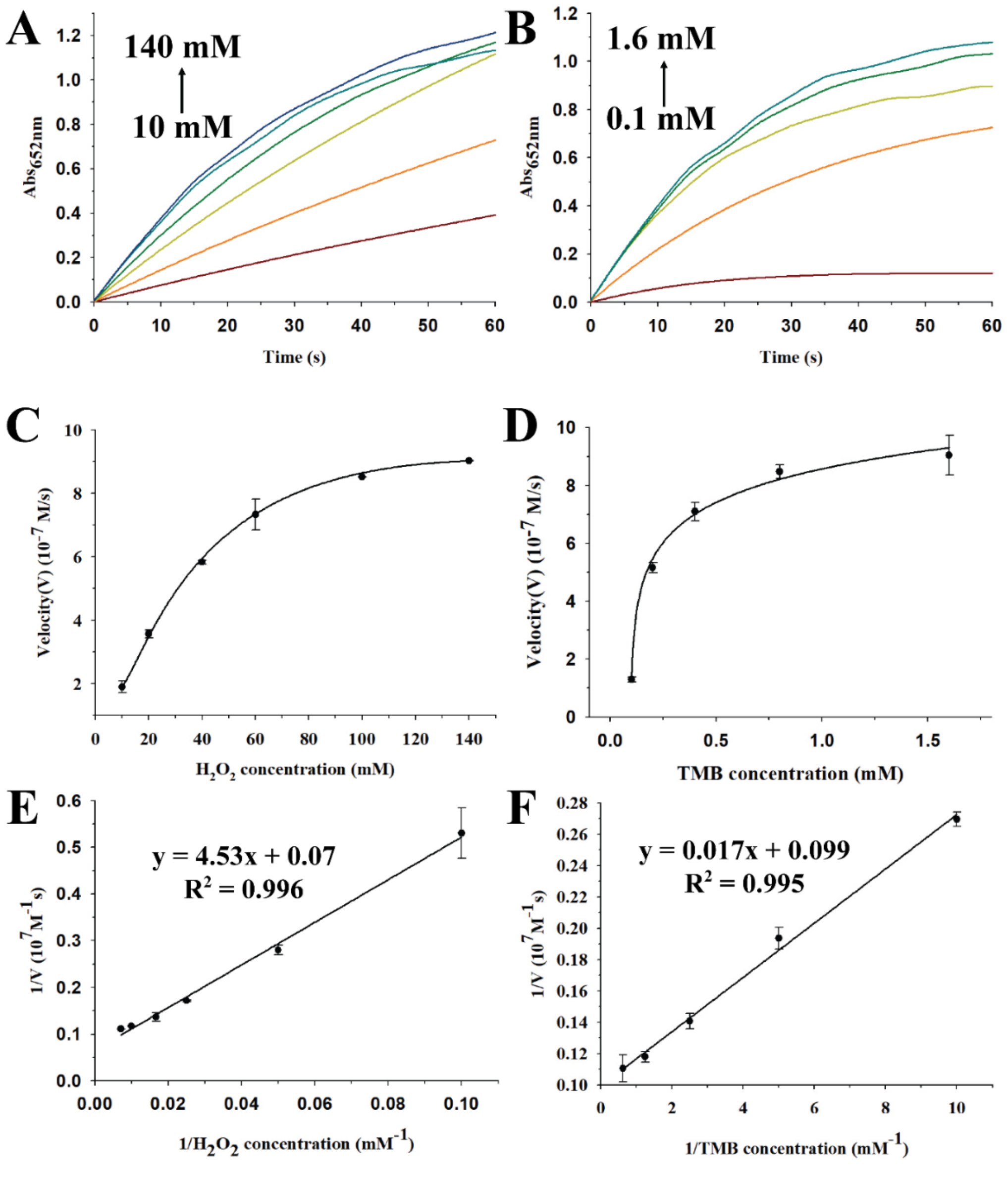

Figure S1. Time-dependent changes in absorbance at 652 nm for different concentrations of (A) H_2O_2 and (B) TMB in the presence of uPtNZs with fixed concentrations of TMB (0.5 mM) and H_2O_2 (100 mM). (C, D) Michaelis-Menten plots and (E, F) Lineweaver-Burk reciprocal plots of H_2O_2 and TMB.

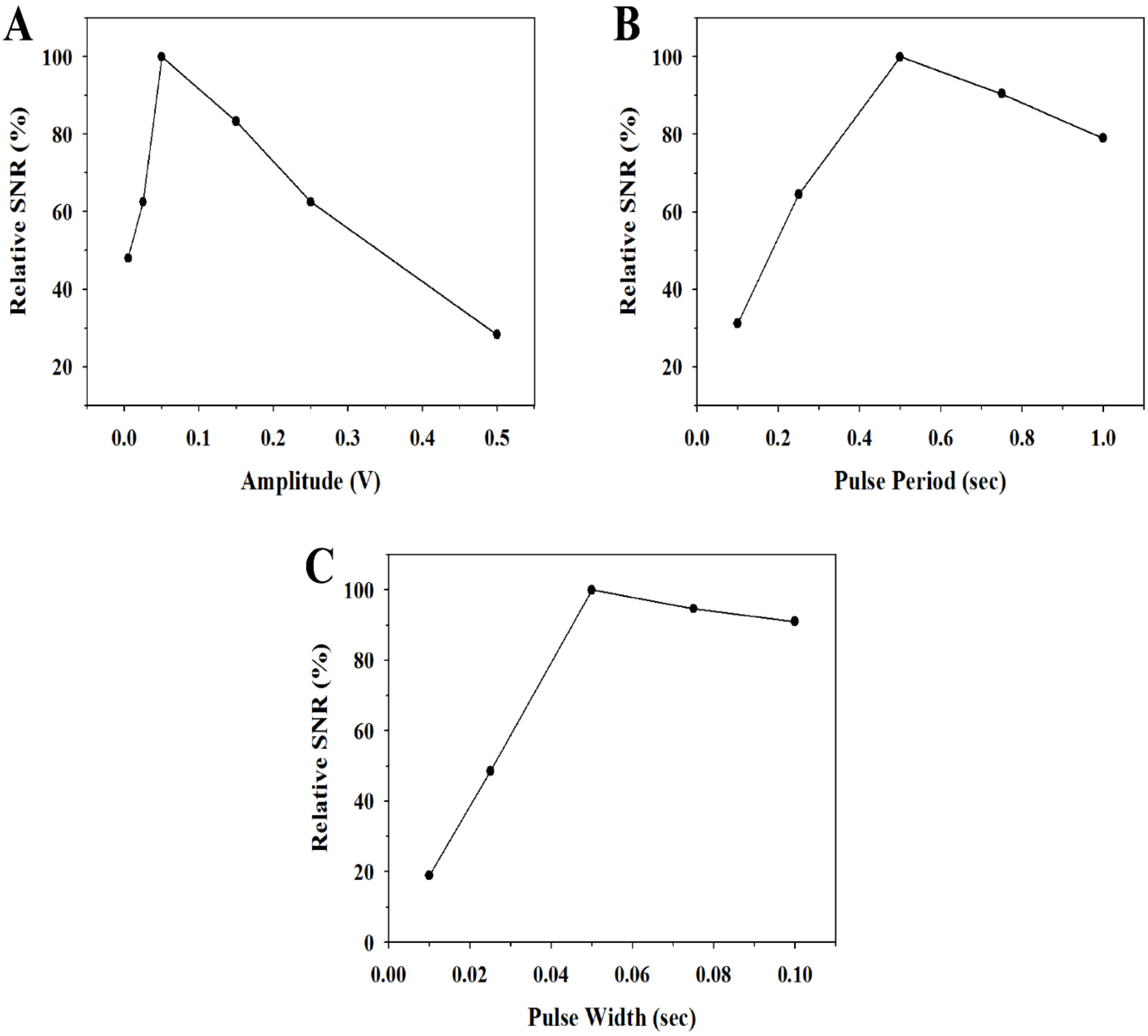

Figure S2. The impacts of (A) amplitude (5 – 500 mV), (B) pulse period (0.01 – 0.1 s), and (C) pulse width (0.1 – 1 s) on the signal to noise ratio (SNR). In each case, other parameters were fixed. The relative SNR (%) refers the ratio of SNR to the highest SNR value obtained from optimized condition.

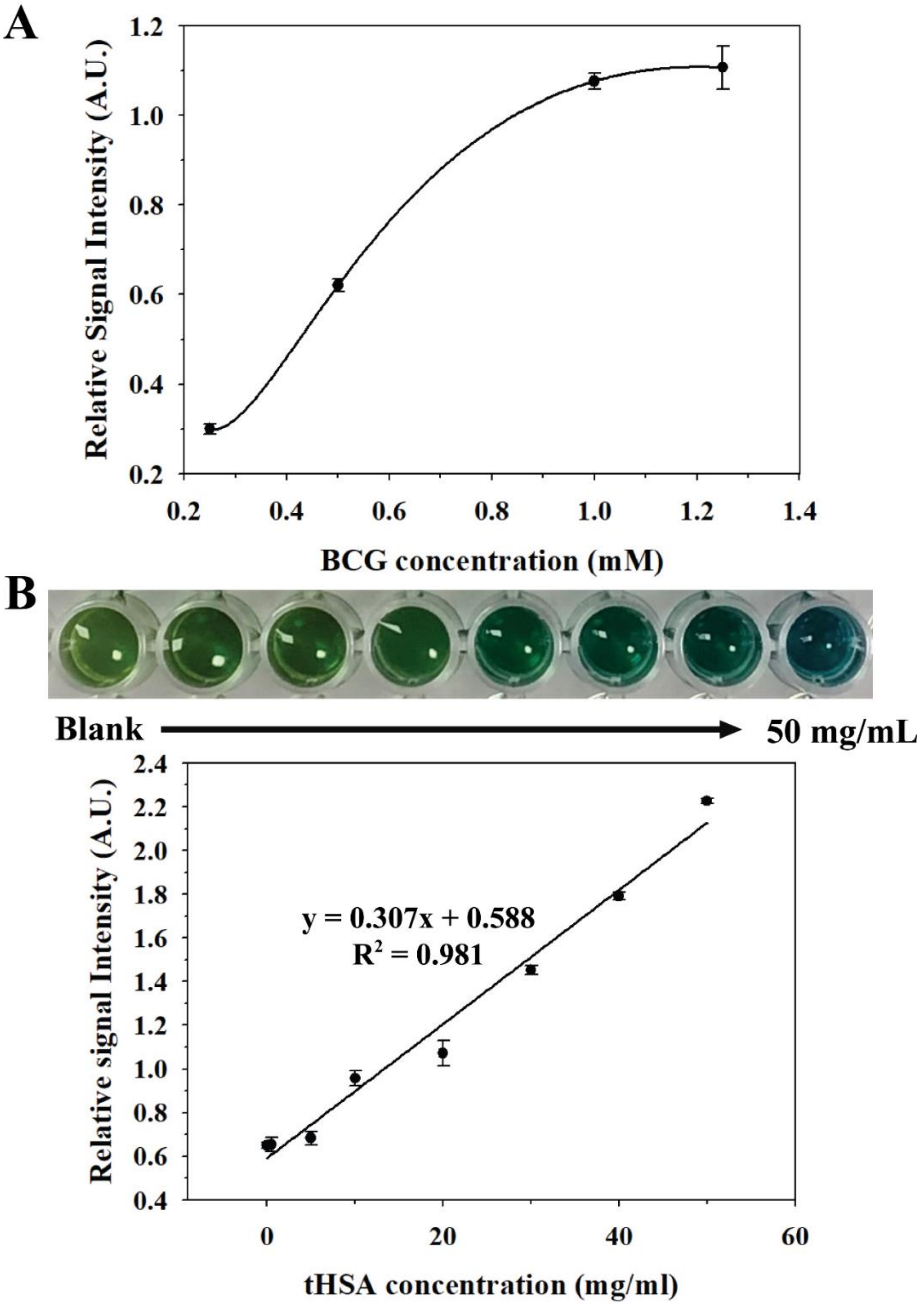

Figure S3. (A) The colorimetric response of 50 mg/mL tHSA (4% GA)-BCG complexes depending on BCG concentration. (B) Photographs of BCG-tHSA complexes at different concentrations of tHSA (blank, 0.5, 5, 10, 20, 30, 40, 50 mg/mL containing 4% GA) and calibration curve for tHSA quantification.

Supplementary Table

Table S1. Comparison of analytical performances with other reported GA determination methods.

Method	LOD (μg/mL)	Linear range (mg/mL)	Ref.
Enzymatic assay based sensor	360	1.2 – 36.8	1
Graphene based optical aptasensor	50	0.05 – 0.3	2
Enzymatic assay based electrochemiluminescence sensor	6.6	–	3
Enzymatic assay based colorimetric sensor	470	5.0 – 7.0	4
Raman spectroscopy-based inspection	465	0.465 – 16.6	5
Colorimetric immunoassay using uPtNZs	9.2	0.01 – 5	This study
Electrochemical immunoassay using uPtNZs	3.8	0.005 – 10	This study

Supplementary References

1 Testa, R. et al. Analytical performances of an enzymatic assay for the measurement of glycated albumin. *J. Appl. Lab. Med.* **1**, 162–171, (2019).

2 Apiwat, C. et al. Graphene based aptasensor for glycated albumin in diabetes mellitus diagnosis and monitoring. *Biosens. Bioelectron.* **82**, 140–145, (2016).

3 Inoue, Y. et al. Sensitive detection of glycated albumin in human serum albumin using electrochemiluminescence. *Anal. Chem.* **89**, 5909–5915, (2017)

4 Kohzuma, T. et al. Basic performance of an enzymatic method for glycated albumin and reference range determination. *J. Diabetes Sci. Technol.* **5**, 1455–1462, (2011).

5 Dingari, N. C. et al. Raman spectroscopy provides a powerful diagnostic tool for accurate determination of albumin glycation. *PLoS One* **7**, e32406, (2012).
